# Comparison of the Effectiveness of Low-Level Laser Therapy and Therapeutic Ultrasound in Patients with Rotator Cuff Tendinopathy

**DOI:** 10.3390/jcm14124197

**Published:** 2025-06-12

**Authors:** Şeyma Diyarbakır, Münevver Serdaroğlu Beyazal, Gül Devrimsel, Murat Yıldırım, Mehmet Serhat Topaloğlu

**Affiliations:** 1 Department of Physical Medicine and Rehabilitation, Agri Training and Research Hospital, 04200 Agri, Turkey; 2 Department of Physical Medicine and Rehabilitation, Faculty of Medicine, Recep Tayyip Erdogan University, 53100 Rize, Turkey

**Keywords:** shoulder pain, rotator cuff, therapeutic ultrasound, laser

## Abstract

**Objectives:** The aims of the presented study were to investigate and compare the effectiveness of Low-Level Laser Therapy (LLLT) and therapeutic ultrasound (US) on pain, function, emotional status, and sleep disturbances in patients with rotator cuff tendinopathy (RCT). **Method:** A total of 84 patients with RCT were included in the study and randomly divided into the US group (*n* = 42) and the LLLT group (*n* = 42). Hot-pack, transcutaneous electrical nerve stimulation, and a home-based exercise program were also administered to patients in each group. The patients were evaluated at baseline, and at 1st, 4th, and 12th weeks after treatment by Visual Analog Scale (VAS), Shoulder Pain and Disability Index (SPADI), Constant Murley Score (CMS), Disabilities of the Arm, Shoulder, and Hand Questionnaire (DASH), Hand Grip Strength (HGS), Beck Depression Inventory (BDI), Beck Anxiety Inventory (BAI), Pittsburgh Sleep Quality Index (PSQI), and Short Form-36 (SF-36). **Results:** Significant improvements in VAS, SPADI, CMS, DASH, BDI, BAI, PSQI, and SF-36 scores were observed over time in both groups (*p* < 0.05 for all). The improvements in HGS scores were significantly greater in the US group compared to the LLLT group (*p* < 0.05 for all). There were no statistically significant differences between the groups in VAS, SPADI, CMS, DASH, BDI, BAI, PSQI, and SF-36 scores at each time point (*p* > 0.05 for all). **Conclusions:** Both therapeutic US and LLLT are effective and safe in the treatment of patients with RCT. However, our findings indicate no superiority of one treatment over the other in terms of pain relief or improvements in function, emotional status, sleep disturbances, or quality of life.

## 1. Introduction

Shoulder pain is one of the common musculoskeletal problems with a prevalence of 7–26% [1,2]. Rotator cuff tendinopathy (RCT), biceps tendon pathologies, acromioclavicular joint pathologies, calcific tendinitis, cervical pathologies, myofascial, and referred pain are among the pathologies causing shoulder pain. RCT affects the individual’s quality of life, and if left untreated, leads to restricted activity and disability in work, education, and social areas. It can develop due to a single traumatic event, overload, and degenerative processes [3]. The research suggests that the diagnoses of subacromial impingement syndrome (SIS), subacromial bursitis, rotator cuff tendonitis, and rotator cuff tears (partial- or full-thickness) arise from tendon degeneration and/or the repetitive or excessive contact of the rotator cuff tendons with other anatomic structures in the shoulder and usually result in functional loss and disability. There are many commonly employed treatments for RCT. Non-steroidal anti-inflammatory drugs (NSAIDs), steroid injections, exercise, manual therapy, physical therapy, and surgical methods are used in the treatment of RCT. Conservative treatment is 40–80% effective in reducing pain and improving function [4].

There are a limited number of studies on the effectiveness of Low-Level Laser Therapy (LLLT) and therapeutic ultrasound (US) in RCT, and conflicting results are obtained among these studies. In some studies, it was thought that LLLT could be used as an alternative to US in RCT, but in others, its superiority over home-based exercise (HBE) could not be demonstrated [5,6,7].

The aims of the presented study were to assess and compare the effectiveness of LLLT and therapeutic US combined with an HBE program on pain, function, emotional status, sleep disturbances, and quality of life in patients with RCT.

## 2. Patients and Methods

**Figure 1:**
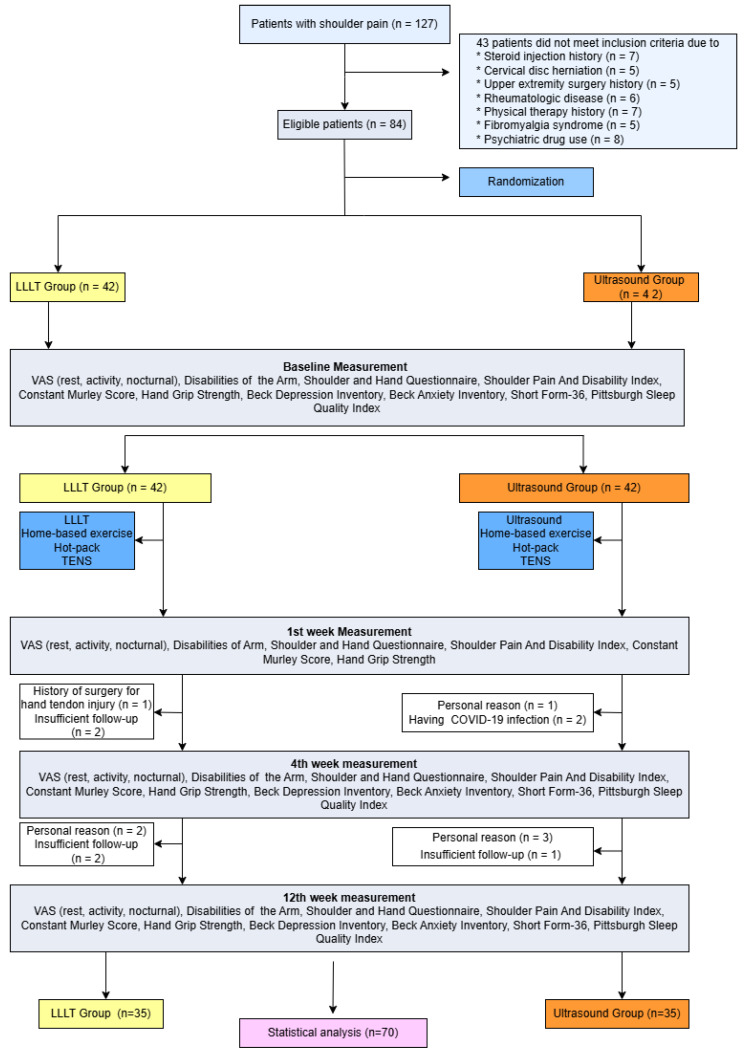
Flowchart of the study. LLLT: Low-Level Laser Therapy, VAS: Visual Analog Scale.

This prospective, observer-blinded, randomized study was conducted at Recep Tayyip Erdogan University, Faculty of Medicine, Department of Physical Medicine and Rehabilitation between April 2021 and February 2022. The study protocol was approved by the local ethics committee of our institution and performed in accordance with the principles stated in the Declaration of Helsinki. All participants provided written informed consent prior to the study. A total of 127 patients were evaluated for compliance with the inclusion criteria of the study. Of these, 84 patients diagnosed with RCT through physical examination and imaging methods were included in the study. The flow diagram of the study is shown in Figure 1. Fourteen participants did not complete the study and were not included in the analysis.

Patients were excluded from the study if they had a history of intra-articular or subacromial injection in the past three months; shoulder surgery or trauma; physical therapy in the past six months, adhesive capsulitis, hand or elbow deformity; radicular pain or cervical disk herniation; malignancy; pregnancy; or psychiatric, metabolic, or inflammatory neuromuscular diseases.

### 2.1. Randomization and Interventions

Randomization was performed using the sealed envelope technique, and patients were allocated into two groups: the US group (*n* = 42) and the LLLT group (*n* = 42). Both LLLT and US were applied over a total of 15 sessions, conducted five times per week. All participants also received a 20 min of hot pack, transcutaneous electrical nerve stimulation (TENS), and an HBE program. The HBE program included Codman pendulum exercises, joint range of motion (ROM) exercises, scapular stabilization exercises, and isometric strengthening exercises. The exercises were prescribed for at least three months, and participants were instructed to perform 10–15 repetitions twice daily.

A therapeutic US device (Chattanooga Medical Supply Inc., Chattanooga, TN, USA) was used for treatment. The US was applied to the painful area of the shoulder in slow circular motions, at a frequency of 1 MHz and an intensity of 1.2 W/cm^2^, using in pulsed mode. The therapy was applied to the painful area of the shoulder using slow circular movements at a frequency of 1 MHz and an intensity of 1.2 W/cm^2^, in pulsed mode. Each session lasted 8 min.

For LLLT, a gallium–aluminum–arsenide (GaAlAs) infrared diode laser device (Chattanooga Medical Supply Inc., Chattanooga, TN, USA) was used, with a wavelength of 850 nm, a power output of 100 mW in continuous wave mode, and a spot area of 0.07 cm^2^. Both patients and the operator wore protective eyeglasses during the procedure. LLLT was administered at a dose of 4 J/cm^2^ to a maximum of five painful points, each for one minute. The total duration of the LLLT session was five minutes per patient.

### 2.2. Outcome Measures

The clinical and sociodemographic characteristics of the patients were recorded. Patients were evaluated with the Visual Analog Scale (VAS), the Disabilities of the Arm, Shoulder, and Hand (DASH), the Shoulder Pain and Disability Index (SPADI), the Constant Murley Score (CMS), the Hand Grip Strength (HGS), the Beck Depression Inventory (BDI), the Beck Anxiety Inventory (BAI), the Pittsburgh Sleep Quality Index (PSQI), and the Short Form-36 (SF-36) at baseline and at the 1st, 4th, and 12th weeks after treatment. Emotional status, sleep disturbances, and quality of life were not evaluated at the 1st week after treatment. Rest, activity, and nocturnal pain intensity were evaluated by a 10 cm horizontal VAS [8,9].

The SPADI consists of 13 items: 5 assess shoulder pain and 8 assess shoulder disability. The total score is converted to a 100-point scale, with higher scores indicating greater impairment. The SPADI is a reliable and highly responsive tool for evaluating shoulder pain and function. Its validity and reliability in the Turkish population were demonstrated by Bumin et al. [10].

The DASH is a 30-item disability/symptom scale that evaluates a patient’s health status and includes 21 items assessing the degree of difficulty in performing various physical activities due to arm, shoulder, or hand problems. Additionally, five items were used to measure the severity of symptoms such as pain, activity-related pain, tingling, weakness, and stiffness. The remaining four items assess the impact of these issues on social activities, work, sleep, and self-image. Each item has five response options. The scores for all items are then used to calculate a scale score ranging from 0 (no disability) to 100 (most severe disability). The validity and reliability of the DASH in the Turkish population were demonstrated by Duger et al. [11].

The CMS consists of four subsections: pain, daily living activities, strength, and ROM. Pain is scored out of 15 points, daily living activities out of 20 points, strength out of 25 points, and ROM out of 40 points, yielding a total score of 100. Higher scores indicate better shoulder function. The validity and reliability of the CMS in the Turkish population were demonstrated by Celik [12].

Hand grip strength was evaluated with the patient seated in a chair, with the elbow flexed at 90 degrees and positioned close to the trunk, and the forearm and wrist in neutral position, using a hand dynamometer. Three measurements were taken, with one-minute rest intervals between them. The results were calculated as the average of the three measurements. Normative reference values have been reported as 40–47 kg for men aged 20–69 (2 kg less in the left hand), 24–30 kg for women aged 20–69 (1.5–2 kg less in the left hand) [13].

The BDI is a 21-item survey that assesses the presence and severity of depression. Higher scores reflect more severe levels of depression. The standard cutoff scores are as follows: 0–9, minimal depression; 10–18, mild depression; 19–29, moderate depression; 30–63, severe depression. The validity and reliability of the BDI in the Turkish population were demonstrated by Hisli [14].

The BAI is relatively brief, easily administered, and scored using a 21-item survey that assesses the severity of anxiety symptoms. The total score ranges from 0 to 63. Scores of 0–9 indicate normal or no anxiety; 10–18, mild to moderate anxiety; 19–29, moderate to severe anxiety; and 30–63, severe anxiety. The validity and reliability of the BAI in the Turkish population were demonstrated by Ulusoy et al. [15].

The PSQI is an 18-item questionnaire that assesses sleep quality and disturbances over a one-month period. It differentiates “poor” sleep from “good” sleep by evaluating seven components, including subjective sleep quality, sleep duration, sleep latency, sleep disturbances, habitual sleep efficiency, daytime dysfunction, and use of sleep medication. A total score of 5 or higher indicates a poor sleep quality. The validity and reliability of the PSQI in the Turkish population were demonstrated by Agargun et al. [16].

The SF-36 is a 36-item questionnaire that measures quality of life across eight domains, covering both physical and emotional aspects of health. These domains are role limitations due to physical health problems (RP), physical functioning (PF), social functioning (SF), bodily pain (BP), general mental health (MH), vitality (VT), role limitations due to emotional problems (RE), and general health perceptions (GH). The validity and reliability of the SF-36 in the Turkish population were previously demonstrated by Demiral et al. [17].

### 2.3. Statistical Analysis

The sample size was calculated using G*Power 3.1. A priori power analysis was conducted with a 5% significance level, 80% power, and medium effect size (dz = 0.5). Based on these assumptions, the required sample size for the study was determined to be 68 patients.

The data obtained from the study were analyzed using the SPSS 24.0 statistical software package. Continuous variables were expressed as mean ± standard deviation (SD). Compliance of the variables with normal distribution was assessed by the Kolmogorov–Smirnov test. Intra-group comparisons were performed using the Friedman and Wilcoxon signed-rank tests. Inter-group analyses were performed with Student’s *t*-test for normally distributed variables and the Mann–Whitney U test for non-normally distributed variables. The chi-square test was used to compare categorical data. To determine the correlation between the variables, Spearman’s rank or Pearson’s correlation analyses were performed according to the distribution of the data. A *p*-value < 0.05 was considered statistically significant, and an adjusted significance level of *p* < 0.001 was applied where appropriate. The effect size for intra-group comparisons was calculated using the Kendall’s W coefficient. For inter-group comparisons, the Mann–Whitney U test was applied, and the effect size was determined using the formula r = z/√n.

## 3. Results

During the follow-up period, fourteen participants did not complete the intervention protocol (Figure 1). These patients were approximately evenly distributed between the two groups (US group: *n* = 7; LLLT group: *n* = 7), and their baseline characteristics did not differ from those who completed the study. A total of 70 patients (mean age 50.8 ± 9.1 years, range 25–65 years) were included in the final analysis. The demographic and clinical characteristics of each group are presented in Table 1. There were no significant differences between groups at baseline in terms of age, gender, BMI, disease duration, pain, HGS, BAI, and PSQI scores (for all, *p* > 0.05). However, the mean BDI scores in the LLLT group were significantly higher compared to those in the US group (12.5 ± 8.8 vs. 7.9 ± 5.9, *p* = 0.010). Additionally, the mean VT, MH, and GH subdomains of SF-36 were significantly higher in the US group than in the LLLT group (*p* = 0.002, *p* = 0.006, *p* = 0.040, respectively).

In both groups, statistically significant increases were observed in the mean CMS, SF-36, and HGS values, and significant decreases were noted in the mean DASH, SPADI, VAS, BDI, BAI, and PSQI scores over time (for all, *p* < 0.05) (Table 2 and Table 3). The improvement in study parameters for each group during the follow-up period is shown in Table 4 and Table 5. There were no significant differences between the US and LLLT groups in VAS, DASH, SPADI, CMS, BDI, BAI, and PSQI scores (*p* > 0.05 for all). However, improvement of the HGS values was significantly greater in the US group compared to the LLLT group at the follow-up period (*p* < 0.05).

## 4. Discussion

The presented study revealed that both LLLT and therapeutic US were effective in reducing pain and improving functional status, emotional well-being, sleep quality, and quality of life in patients with RCT. However, therapeutic US demonstrated better improvement in HGS compared to LLLT.

Rotator cuff tendinopathy is one of the most common musculoskeletal disorders. RCT encompasses a range of pathologies, including bursitis, SIS, tendonitis, and partial- or full-thickness rotator cuff tears. Traditional treatment of RCT involves a variety of therapies, such as administration of analgesics and anti-inflammatory drugs, application of hot and cold packs, TENS, acupuncture, exercise, manual therapy, subacromial corticosteroid injections, nutritional supplements, as well as subacromial decompression and/or surgical repair of rotator cuff tears [18].

Therapeutic US is commonly used in the conservative treatment of RCT and is often prescribed in combination with other interventions. The physiologic effects of US are increased vascular permeability, blood flow, local metabolism, and increased fibrous tissue extensibility and muscle relaxation [19]. Several studies have reported conflicting findings regarding the efficacy of therapeutic US as a physical therapy modality [20]. Desmeules et al. reported that therapeutic US did not provide any significant benefit when compared to placebo, laser therapy, or when combined with exercise [21]. In our study, both therapeutic US and LLLT improved all clinical parameters, with greater improvement in HGS observed in the US group compared to the LLLT group during the follow-up period. Also, Yildirim et al. [22] reported that therapeutic US improved pain and functional status in patients with SIS. Similarly to our study, they also evaluated emotional status using the BDI. In addition, our study assessed HGS, anxiety, sleep disturbances, and quality of life.

The physiologic effects of the LLLT include increased collagen synthesis, cell proliferation, and blood flow, as well as promotion of tissue regeneration and reduction in inflammation [23]. Tombak et al. reported that HBE combined with LLLT for the rotator cuff calcific tendinitis was more effective than HBE alone in improving pain and disability [24]. In our study, we evaluated strength and ROM using the CMS scale. Additionally, we assessed HGS, emotional status, sleep disturbances, and quality of life. A systematic review also reported that LLLT is used as a treatment for SIS [25]. However, it has been advised that the benefits of LLLT should be further investigated in future studies. Atıcı et al. found that LLLT is not superior to placebo in the treatment of SIS [26]. In their study, LLLT was applied at a dosage of 4 joules, similar to our protocol. On the other hand, the treatment duration differed from that in our study. Additionally, our study included a longer follow-up period and further assessment, such as strength using CMS, emotional status, sleep disturbances, and quality of life.

In the present study, the efficacy of therapeutic US and LLLT was compared in patients with RCT, and no significant differences were observed between groups in terms of pain relief, functional improvement, emotional status, sleep disturbances, or quality of life. However, there was a significant baseline difference in BDI scores and some subdomains of the SF-36 between the groups. To account for these imbalances, inter-group comparisons were conducted using change scores (i.e., the difference between baseline and post-treatment values) rather than absolute values. This approach allowed for the evaluation of treatment effects while minimizing the potential bias introduced by baseline differences. Our findings were consistent with those of previous studies [5,6,7,27]. Yavuz et al. demonstrated that LLLT and therapeutic US improved pain, disability, and sleep disturbances in SIS, and suggested that LLLT could be used when US is contraindicated [5]. Unlike our study, that investigation included a smaller patient cohort. Calis et al. also reported that both LLLT and therapeutic US improved pain, function, and ROM in patients with SIS, although no significant differences were observed between the two modalities [6]. Our study differed in that it included a longer follow-up period and a more comprehensive evaluation of patient outcomes. In addition to pain, function, and ROM, we also assessed HGS, sleep disturbances, emotional status, and quality of life, and observed improvements across all parameters in both the LLLT and US groups. Sen et al. reported that both LLLT and therapeutic US, when combined with an HBE program, led to significant improvements in pain and disability in patients with SIS [7]. However, in contrast to our findings, they reported that therapeutic US had no additional benefit in the short term. In their study, LLLT combined with HBE was more effective than HBE alone in relieving activity pain and improving shoulder function in the short term. In contrast to the study by Sen et al., the absence of an HBE-only group in our study constitutes a limitation, as it precludes a clear assessment of the specific contribution of HBE compared to the active modalities (US or LLLT). In our study, therapeutic US not only improved pain and disability but also led to greater improvements in HGS compared to LLLT, and was effective across all measured parameters. Importantly, our study incorporated additional outcome measures that are often overlooked. These included emotional status, sleep disturbance, and quality of life, along with the use of the CMS to evaluate shoulder strength. The follow-up period was similar to that of Sen et al., yet our broader outcome assessment provides a more comprehensive evaluation of treatment efficacy. A study by Ranjithkumar et al. found that low-energy extra-corporeal shockwave therapy (ESWT), LLLT, and US are effective treatments for RCT, while ESWT is more effective in improving muscle thickness, shoulder function, and pain level [27]. Unlike our study, Saunders reported that laser treatment was significantly more effective in improving muscle force than US [28]. In addition, Santamato et al. also reported that high-intensity laser therapy was more effective than US therapy in reducing pain and improving joint mobility, functional status, and muscle strength in patients with SIS short-term follow-up period [29]. The discrepancies among studies may be attributed to differences in study design, sample size, treatment protocols, follow-up durations, and the range of outcome measures assessed.

This study has several limitations that should be considered when interpreting the findings. First, the absence of a placebo or control group limits the ability to determine the isolated effects of LLLT and US therapy. Second, the lack of blinding among patients, due to the nature of the interventions, may have introduced bias. Third, although the follow-up period was longer than in some prior studies, it may still be insufficient to assess long-term outcomes.

## 5. Conclusions

In conclusion, this study demonstrated that both therapeutic US and LLLT were effective in the management of RCT, leading to significant improvements in pain, functional status, emotional status, sleep quality, and quality of life. Notably, therapeutic US led to greater improvements in HGS compared to LLLT. These findings contribute to the growing body of evidence supporting the use of both modalities in the treatment of RCT. Therapeutic US may be preferred in cases where improving HGS is a clinical priority. However, due to limitations such as the lack of a placebo group and the single-center design, further randomized controlled trials with larger sample sizes and placebo-controlled protocols are warranted to better define the optimal treatment approach.

## Figures and Tables

**Table 1:** Baseline demographic and clinical characteristics of participants in the US and LLLT groups.

	Ultrasound Group (*n* = 35)	LLLT Group (*n* = 35)	*p*-Value
Age, years (mean ± SD)	51.7 ± 8.8	49.9 ± 9.4	0.410
Gender, F/M (*n*, %)	20/15(57.1/42.9)	20/15 (57.1/42.9)	1.000
BMI (mean ± SD)	30.4 ± 5.8	29.1 ± 4.9	0.310
Disease duration, months (mean ± SD)	13.6 ± 16.2	9.4 ± 1.2	0.210
Side of involvement (R/L), *n* (%)	23/12 (65.7/34.3)	24/11 (68.6/31.4)	0.790
Occupation, *n* (%)			0.730
Housewife	18 (51.4)	13 (37.2)	
Retired	4 (11.4)	6 (17.1)	
Active employee	13 (37.2)	16 (45.7)	
VAS (mean ± SD)			
Activity	8.3 ± 1.5	8.6 ± 0.8	0.330
Rest	5.4 ± 0.8	5.4 ± 0.9	0.680
Night	6.9 ± 1.9	7.6 ± 1.5	0.170
DASH (mean ± SD)			
Function-symptom	51.8 ± 15.6	50.3 ± 16.9	0.714
Work	64.1 ± 20.9	55.3 ± 20.5	0.870
CMS (mean ± SD)			
Total	60.5 ± 7.6	58.4 ± 4.7	0.170
Objective assessment	49.5 ± 5.7	49.1 ± 3.7	0.747
SPADI (mean ± SD)			
Pain	80.8 ± 17.3	77.5 ± 10.9	0.342
Disability	68.6 ± 19.1	63.5 ± 13.1	0.202
Total	73.3 ± 17.7	68.9 ± 11.6	0.225
HGS, kg (mean ± SD)			
Right	28.6 ± 9.6	29.6 ± 10.5	0.600
Left	28.1 ± 9.2	30.2 ± 9.7	0.300
SF-36 (mean ± SD)			
Physical functioning	63.3 ± 16.93	60.3 ± 21.5	0.500
Role physical	17.9 ± 33.52	33.6 ± 39.7	0.070
Role emotional	56.2 ± 44.11	49.5 ± 39.9	0.500
Vitality	64.1 ± 20.38	48.6 ± 20.3	0.002 *
Mental health	69.3 ± 14.41	58.9 ± 15.9	0.006 *
Social functioning	67.5 ± 25.4	63.6 ± 20.4	0.470
Bodily pain	34.8 ± 23.1	33.7 ± 20.9	0.840
General Health	55.9 ± 16.3	48.0 ± 16.4	0.040 *
BDI (mean ± SD)	7.9 ± 5.9	12.5 ± 8.8	0.010 *
BAI (mean ± SD)	13.1 ± 11.6	14.2 ± 8.5	0.600
PSQI (mean ± SD)	7.3 ± 3.9	7.6 ± 3.3	0.700

LLLT, Low-Level Laser Therapy; US, Ultrasound; VAS, Visual Analog Scale; DASH, Disabilities of the Arm, Shoulder and Hand; CMS, Constant Murley Score; SPADI, Shoulder Pain and Disability Index; HGS, Hand Grip Strength; SF-36, Short Form-36; BDI, Beck Depression Inventory; BAI, Beck Anxiety Inventory; PSQI, Pittsburgh Sleep Quality Index; *, statistically significant.

**Table 2:** Intra-group comparison of the clinical characteristics over follow-up in the therapeutic ultrasound group.

Ultrasound Group (*n* = 35)	Baseline	Post-Treatment 1st Week	Post-Treatment 4th Week	Post-Treatment 12th Week	*p*-Value *	Effect Size (Kendall’s W)
DASH						
Function Symptom	51.8 ± 15.6	34.8 ± 21.9	28.1 ± 18.8	15.1 ± 17.5	<0.001 **	0.665
Work	64.1 ± 20.9	45.9 ± 25.7	39.5 ± 24.3	19.7 ± 22.2	<0.001 **	0.586
SPADI						
Pain	80.8 ± 17.3	47.5 ± 24.5	43.1 ± 30.2	25.6 ± 25.9	<0.001 **	0.691
Disability	68.6 ± 19.1	41.1 ± 27.6	35.0 ± 24.0	18.0 ± 20.6	<0.001 **	0.609
Total	73.3 ± 17.7	43.0 ± 25.9	38.2 ± 26.1	20.3 ± 22.2	<0.001 **	0.717
CMS						
Total	60.5 ± 7.6	80.0 ± 13.8	88.9 ± 11.5	96.4 ± 5.2	<0.001 **	0.806
Objective Assessment	49.5 ± 5.7	58.9 ± 7.0	61.9 ± 5.7	64.5 ± 1.2	<0.001 **	0.733
VAS						
Activity	8.3 ± 1.5	5.8 ± 2.4	4.5 ± 1.9	2.5 ± 2.3	<0.001 **	0.783
Rest	5.4 ± 0.8	3.0 ± 2.1	1.1 ± 1.7	0.5 ± 1.2	<0.001 **	0.777
Night	6.9 ± 2.0	3.0 ± 2.7	1.0 ± 1.8	0.9 ± 1.5	<0.001 **	0.788
HGS						
Right	28.6 ± 9.6	32.2 ± 9.8	31.5 ± 9.9	33.5 ± 9.4	<0.001 **	0.248
Left	28.2 ± 9.2	32.2 ± 8.6	31.9 ± 9.3	33.1 ± 9.3	<0.001 **	0.306
BDI	7.9 ± 6.0	x	5.4 ± 5.2	5.0 ± 6.5	<0.001 **	0.276
BAI	13.1 ± 11.6	x	8.3 ± 7.3	8.1 ± 9.0	0.002 **	0.181
PSQI	7.3 ± 3.9	x	4.5 ± 3.7	3.1 ± 3.4	<0.001 **	0.325
SF-36						
Physical Functioning	63.3 ± 16.9	x	78.4 ± 15.2	77.6 ± 20.3	<0.001 **	0.290
Role Physical	17.9 ± 33.5	x	60.7 ± 42.1	64.3 ± 43.0	<0.001 **	0.394
Role Emotional	56.2 ± 44.1	x	84.8 ± 31.7	75.2 ± 39.9	0.001 **	0.205
Vitality	64.1 ± 20.4	x	70.7 ± 18.2	69.1 ± 18.0	0.074	0.075
Mental Health	69.3 ± 14.4	x	73.8 ± 12.8	72.8 ± 15.6	0.021 **	0.110
Social Functioning	67.5 ± 25.4	x	80.2 ± 21.3	84.6 ± 19.5	0.001 **	0.217
Bodily Pain	34.8 ± 23.1	x	58.6 ± 26.1	69.2 ± 26.5	<0.001 **	0.527
General Health	55.9 ± 16.3	x	67.3 ± 12.7	70.0 ± 22.5	<0.001 **	0.375

DASH, Disabilities of the Arm, Shoulder and Hand; SPADI, Shoulder Pain and Disability Index; VAS, Visual Analog Scale; CMS, Constant Murley Score; HGS, Hand Grip Strength; BDI, Beck Depression Inventory; BAI, Beck Anxiety Inventory; PSQI, Pittsburgh Sleep Quality Index; SF-36, Short Form-36; *, *p*-values are based on the Friedman Test; x, not assessed at this time point; **, statistically significant.

**Table 3:** Intra-group comparison of the clinical characteristics over follow-up in the LLLT group.

LLLT Group (*n* = 35)	Baseline	Post-Treatment 1st Week	Post-Treatment 4th Week	Post-Treatment 12th Week	*p*-Value *	Effect Size (Kendall’s W)
DASH						
Function Symptom	50.4 ± 16.9	30.4 ± 17.8	29.5 ± 22.5	16.4 ± 19.5	<0.001 **	0.530
Work	55.4 ± 20.8	37.5 ± 21.0	28.6 ± 24.0	16.1 ± 24.9	<0.001 **	0.508
SPADI						
Pain	77.5 ± 10.9	47.5 ± 20.8	43.3 ± 30.0	25.1 ± 28.9	<0.001 **	0.554
Disability	63.5 ± 13.1	41.9 ± 19.4	35.8 ± 24.9	23.2 ± 26.6	<0.001 **	0.544
Total	68.9 ± 11.6	45.6 ± 19.0	38.7 ± 26.7	23.9 ± 27.1	<0.001 **	0.559
CMS						
Total	58.4 ± 4.7	81.8 ± 10.3	86.7 ± 9.9	95.4 ± 8.8	<0.001 **	0.820
Objective Assessment	49.1 ± 3.7	61.2 ± 5.0	63.2 ± 3.5	64.3 ± 2.0	<0.001 **	0.790
VAS						
Activity	8.6 ± 0.8	5.7 ± 1.8	4.6 ± 2.0	2.7 ± 2.4	<0.001 **	0.884
Rest	5.5 ± 1.0	2.9 ± 1.4	2.0 ± 1.8	0.9 ± 1.4	<0.001 **	0.785
Night	7.6 ± 1.5	3.5 ± 2.3	2.3 ± 2.1	1.1 ± 1.9	<0.001 **	0.853
HGS (kg)						
Right	29.6 ± 10.5	30.1 ± 9.3	30.9 ± 8.7	33.1 ± 9.0	<0.001 **	0.243
Left	30.2 ± 9.8	31.1 ± 9.9	31.1 ± 8.7	32.5 ± 8.9	<0.001 **	0.117
BDI	12.5 ± 8.8	x	8.8 ± 9.1	8.4 ± 7.9	<0.001 **	0.235
BAI	14.2 ± 8.5	x	10.6 ± 8.4	9.4 ± 8.5	<0.001 **	0.319
PSQI	7.6 ± 3.3	x	5.4 ± 4.2	4.9 ± 3.9	<0.001 **	0.245
SF-36						
Physical Functioning	60.3 ± 21.5	x	74.0 ± 19.7	77.4 ± 18.4	0.001 **	0.210
Role Physical	33.6 ± 39.7	x	64.3 ± 39.9	74.3 ± 36.6	<0.001 **	0.342
Role Emotional	49.5 ± 39.9	x	66.7 ± 36.2	81.0 ± 32.6	0.001 **	0.207
Vitality	48.6 ± 20.3	x	63.4 ± 16.2	64.3 ± 23.6	<0.001 **	0.321
Mental Health	58.9 ± 15.9	x	66.3 ± 14.0	70.7 ± 16.3	0.001 **	0.213
Social Functioning	63.6 ± 20.4	x	75.4 ± 21.2	83.5 ± 17.6	<0.001 **	0.337
Bodily Pain	33.7 ± 21.0	x	63.3 ± 27.3	74.4 ± 25.3	<0.001 **	0.600
General Health	48.0 ± 16.4	x	61.1 ± 22.9	65.3 ± 18.2	<0.001 **	0.274

**Table 4:** Inter-group comparison of score changes in VAS, DASH, SPADI, CMS, and HGS over time.

	Ultrasound Group (*n* = 35)	LLLT Group (*n* = 35)	*p*-Value	Effect Size (r)
VAS:				
Activity				
Baseline vs. W1	−2.5 ± 2.8	−2.9 ± 1.7	0.280	0.128
Baseline vs. W4	−3.8 ± 2.4	−4.0 ± 1.9	0.910	0.013
Baseline vs. W12	−5.8 ± 2.6	−5.9 ± 2.3	0.980	0.002
Rest				
Baseline vs. W1	−2.4 ± 2.0	−2.6 ± 1.4	0.620	0.059
Baseline vs. W4	−4.3 ± 1.7	−3.5 ± 2.0	0.050	0.232
Baseline vs. W12	−4.9 ± 1.1	−4.6 ± 1.6	0.410	0.098
Night				
Baseline vs. W1	−3.9 ± 2.5	−4.1 ± 2.0	0.600	0.062
Baseline vs. W4	−5.9 ± 2.3	−5.3 ± 2.1	0.240	0.139
Baseline vs. W12	−6.2 ± 2.1	−6.5 ± 1.8	0.530	0.074
DASH:				
Function-symptom				
Baseline vs. W1	−17.0 ± 16.7	−20.0 ± 17.9	0.660	0.053
Baseline vs. W4	−23.7 ± 15.0	−20.8 ± 20.4	0.390	0.101
Baseline vs. W12	−36.7 ± 17.9	−34.0 ± 23.7	0.550	0.070
Work				
Baseline vs. W1	−18.2 ± 21.6	−18.2 ± 22.3	0.850	0.022
Baseline vs. W4	−24.6 ± 19.9	−27.0 ± 20.3	0.510	0.078
Baseline vs. W12	−44.4 ± 25.5	−39.3 ± 26.9	0.520	0.077
SPADI:				
Pain				
Baseline vs. W1	−33.3 ± 22.7	−30.0 ± 20.7	0.484	0.084
Baseline vs. W4	−37.7 ± 25.8	−34.1 ± 30.4	0.742	0.039
Baseline vs. W12	−55.2 ± 23.0	−52.4 ± 29.7	0.930	0.010
Disability				
Baseline vs. W1	−27.4 ± 26.3	−21.6 ± 18.5	0.470	0.086
Baseline vs. W4	−33.6 ± 21.6	−27.7 ± 24.4	0.350	0.109
Baseline vs. W12	−50.6 ± 21.2	−40.3 ± 26.8	0.100	0.192
Total				
Baseline vs. W1	−30.3 ± 24.8	−23.3 ± 17.8	0.260	0.132
Baseline vs. W4	−35.1 ± 22.5	−30.2 ± 26.1	0.400	0.100
Baseline vs. W12	−53.0 ± 21.1	−45.0 ± 27.4	0.290	0.125
CMS:				
Total				
Baseline vs. W1	19.5 ± 13.2	23.4 ± 9.6	0.120	0.183
Baseline vs. W4	28.5 ± 12.3	28.3 ± 9.8	0.710	0.044
Baseline vs. W12	35.9 ± 7.5	37.0 ± 9.2	0.240	0.138
CMS				
Objective assessment				
Baseline vs. W1	9.4 ± 6.8	12.1 ± 5.1	0.050	0.229
Baseline vs. W4	12.5 ± 6.7	14.1 ± 4.2	0.320	0.117
Baseline vs. W12	46.9 ± 6.2	46.3 ± 9.2	0.540	0.072
HGS				
Right				
Baseline vs. W1	3.7 ± 4.7	0.4 ± 5.5	0.010 **	0.306
Baseline vs. W4	3.0 ± 5.1	1.2 ± 6.4	0.370	0.107
Baseline vs. W12	4.9 ± 5.4	3.5 ± 7.3	0.460	0.088
Left				
Baseline vs. W1	4.0 ± 5.0	0.9 ± 3.7	0.007 **	0.320
Baseline vs. W4	3.7 ± 5.1	0.8 ± 4.3	0.040 **	0.240
Baseline vs. W12	4.9 ± 4.9	2.2 ± 5.0	0.030 **	0.252

LLLT, Low-Level Laser Therapy; VAS, Visual Analog Scale; DASH, Disabilities of the Arm, Shoulder, and Hand; SPADI, Shoulder Pain and Disability Index; CMS, Constant Murley Score; HGS, Hand Grip Strength; W1, 1st week post-treatment; W4, 4th week post-treatment; W12, 12th week post-treatment; **, statistically significant.

**Table 5:** Inter-group comparison of score changes in SF-36, BDI, BAI, and PSQI over time.

	US Group (*n* = 35)	LLLT Group (*n* = 35)	*p* Value	Effect Size (r)
SF-36:				
Physical functioning				
Baseline vs. W4	15.1 ± 1.6	13.7 ± 18.2	0.870	0.019
Baseline vs. W12	14.3 ± 24.7	17.1 ± 25.7	0.760	0.037
Role Physical				
Baseline vs. W4	42.9 ± 44.0	30.7 ± 43.3	0.230	0.142
Baseline vs. W12	46.4 ± 45.8	40.7 ± 43.8	0.560	0.068
Role emotional				
Baseline vs. W4	28.6 ± 39.7	17.1 ± 54.5	0.480	0.083
Baseline vs. W12	19.0 ± 41.4	31.4 ± 42.7	0.130	0.181
Vitality				
Baseline vs. W4	6.5 ± 11.5	14.9 ± 21.9	0.070	0.210
Baseline vs. W12	5.0 ± 22.1	15.7 ± 21.2	0.040	0.238
Mental health				
Baseline vs. W4	4.6 ± 12.2	7.4 ± 15.5	0.640	0.055
Baseline vs. W12	3.5 ± 18.2	11.9 ± 16.9	0.160	0.166
Social functioning				
Baseline vs. W4	12.7 ± 18.4	11.8 ± 24.3	0.880	0.017
Baseline vs. W12	17.1 ± 32.5	19.9 ± 22.3	0.860	0.021
Bodily pain				
Baseline vs. W4	45.4 ± 26.1	41.6 ± 23.6	0.560	0.070
Baseline vs. W12	34.4 ± 31.5	40.7 ± 31.7	0.480	0.083
General Health				
Baseline vs. W4	11.3 ± 13.5	13.1 ± 21.1	0.830	0.025
Baseline vs. W12	14.1 ± 21.6	17.3 ± 19.5	0.800	0.030
BDI				
Baseline vs. W4	−2.5 ± 5.2	−3.7 ± 7.1	0.290	0.124
Baseline vs. W12	−2.9 ± 6.2	−4.1 ± 6.7	0.670	0.049
BAI				
Baseline vs. W4	−4.7 ± 8.9	−3.6 ± 5.8	0.860	0.021
Baseline vs. W12	−4.9 ± 12.8	−4.9 ± 6.5	0.850	0.022
PSQI				
Baseline vs. W4	−2.8 ± 3.4	−2.2 ± 3.9	0.490	0.081
Baseline vs. W12	−4.2 ± 4.5	−2.6 ± 4.1	0.140	0.173

LLLT, Low-Level Laser Therapy; SF-36, Short Form-36; BDI, Beck Depression Inventory; BAI, Beck Anxiety Inventory; PSQI, Pittsburgh Sleep Quality Index; W1, 1st week post-treatment; W4, 4th week post-treatment; W12, 12th week post-treatment.

## Data Availability

The data presented in this study are available on request from the corresponding author.
